# Opioid use disorder from poppy seed tea successfully treated with buprenorphine in primary care: a case report

**DOI:** 10.1186/s13722-021-00280-4

**Published:** 2021-12-03

**Authors:** Scott Hagan, Carol E. Achtmeyer, Carly Hood, Eric J. Hawkins, Emily C. Williams

**Affiliations:** 1 Department of Medicine, University of Washington School of Medicine, Seattle, WA USA; 2 General Medicine Service, Veterans Affairs (VA) Puget Sound Health Care System Seattle Division, Seattle, WA USA; 3 Center of Excellence in Substance Addiction Treatment and Education, Veterans Affairs (VA) Puget Sound Health Care System Seattle Division (S116ATC), 1660 S. Columbian Way, Seattle, WA USA; 4 Health Services Research & Development (HSR&D), Seattle Center of Innovation for Veteran-Centered and Value-Driven Care, Veterans Affairs (VA) Puget Sound Health Care System, Seattle, WA USA; 5 Department of Psychiatry and Behavioral Sciences, University of Washington School of Medicine, Seattle, WA USA; 6 Department of Health Systems and Population Health, University of Washington School of Public Health, Seattle, WA USA

**Keywords:** Opioid use disorder, Poppy seed tea, Buprenorphine

## Abstract

**Background:**

Poppy seeds contain morphine and other opioid alkaloids and are commercially available in the United States. Users of poppy seed tea (PST) can consume several hundred morphine milligram equivalents per day, and opioid dependence from PST use can develop. We report a case of a patient with chronic pain and PST use leading to opioid use disorder (OUD). This case represents the first published report of OUD from PST successfully treated with buprenorphine (BUP) in a primary care setting. The provider in this case used a unique model of care with an opioid prescribing support team to deliver safe and effective care.

**Case presentation:**

A 47-year-old man with chronic pain and prescription opioid use presented to primary care to discuss a flare of shoulder pain, and revealed in subsequent conversation a long-standing use of PST to supplement pain control. Attempts at cessation resulted in severe withdrawal symptoms, leading to return to PST use. The primary care provider consulted the VA Puget Sound **SU**pporting **P**rimary care **P**roviders in **O**pioid **R**isk reduction and **T**reatment (SUPPORT) team to evaluate the patient for OUD. The patient discontinued all opioids, and initiated BUP under the supervision of the primary care provider. He remained on a stable dosage, without relapse, 24 months later.

**Conclusions:**

PST, which can be made through purchase of readily available poppy pods, carries risk for development of OUD and overdose. Herein we highlight the utility of a primary care opioid prescribing support team in empowering a primary care provider to prescribe BUP to treat a patient with complex OUD.

## Background

*Papaver somniferum*, also known as opium poppy, is a flowering plant whose seed pods contain numerous opioid alkaloids. Poppy seed pods are commercially available from online and in-store vendors in the United States, but the prevalence of purchase for use in poppy seed tea (PST) concoctions is unknown. The main active opioid alkaloids in PST are morphine, codeine, and thebaine (Table 1). Although the concentration of these substances is highly variable depending on the product and the extraction technique [1], users of PST can consume several hundred daily morphine milligram equivalents (MME) [2], increasing risk for opioid overdose and OUD.

**Table 1:** Mean opioid concentrations in poppy seed tea

Opioid Alkaloid	Concentration (mg alkaloid per kg seeds)	Estimated Dose (mg alkaloid per 500 mL tea)
Morphine	352–481	100–136
Codeine	51–68	14–19
Thebaine	23–45	N/A^a^

Ranges of mean concentration and estimated dose of 22 different commercially available poppy seed products tested using four different extraction techniques [1]. The estimated dose assumes an average use of 283 g per 500 mL [1]

^a^The analgesic or psychoactive properties of thebaine are unknown [3]

Despite legislation enabling primary care providers to prescribe medications for OUD (MOUD) since 2002, treatment for OUD has been difficult to scale in primary care. Consistent evidence suggests that most people diagnosed with OUD never receive any form of treatment [4]. While there are prior case reports of patients with OUD from PST receiving MOUD through addiction treatment programs, there are no known cases of MOUD initiation and maintenance for this indication in a primary care clinic (PCC). Increasing public pressure may soon lead to increased regulation of the poppy pod industry in the U.S. as it has in other countries [5], and as a result of decreased access, patients using PST may seek evaluations in primary care for OUD-related symptoms.

## Case presentation

A 47-year-old United States veteran with post-traumatic stress disorder (PTSD) and 15-year history of low back, knee, neck, and shoulder pain presented to PCC to discuss pain management. Two weeks prior to the appointment, his primary care provider (PCP) diagnosed a flare of proximal biceps tendinopathy as the cause of worsening right shoulder pain and recommended a course of physical therapy. After reviewing this condition, the patient asked to discuss his opioid contract. He started chronic prescription opioid therapy 12 years ago when other treatments including physical therapy, acetaminophen, piroxicam, gabapentin, and amitriptyline provided insufficient pain relief. Prior opioid regimens included fentanyl, hydrocodone, hydromorphone, morphine, and tramadol. The maximum daily MME was 422 from the combination of a fentanyl patch and hydromorphone tablets prescribed eight years ago in a pain clinic. The following year, pain clinic providers concluded that the patient’s undertreated PTSD with accompanying anxiety was significantly exacerbating his pain, and that he was insufficiently engaged in recommended cognitive behavioral therapy for chronic pain. Therefore, they initiated a taper with the goal to discontinue the opioid regimen. However, due to significantly increased pain during the taper, prescriptions for opioids continued at an MME below 90. At the time of the PCC appointment, he had been taking 10 mg three times per day (TID) of oxycodone for two years since establishing care with his current PCP.

During our visit, his pain control was adequate, but he shared, for the first time, that he had been using PST to supplement pain control for the past 7 years. He bought poppy pods at farmer’s markets and online retailers and would prepare a hot tea from grounds of the pods. Depending on his perceived analgesic effect from the tea, he drank 2–4 cups per day, using five large poppy pods per tea serving. The average monthly cost of the PST, which increased substantially in the past 5 years for him, was $2000. He did not have psychoactive effects from the tea; however, he did have prolonged withdrawal symptoms when he made attempts to discontinue use. His withdrawal symptoms were shaking chills, intense pain, headache, yawning and diarrhea. These symptoms presented within 24 h (h) of abstinence, and would intensify over several days, always leading him to return to PST use. He bought poppy pods through legal sources and never used illicit opioids.

He had been reluctant to report PST use to clinicians for fear of discontinuation of his opioid prescription. In retrospect, his PCP noted that prior annual urine drug screens (UDS) were consistently positive for opiates, which at the PCC’s laboratory is a separate assay from oxycodone, indicating that he had consumed another type of opioid (e.g., morphine or codeine from PST). The patient desired complete discontinuation of opioids, both prescription medications and PST, because he was frustrated with opioid dependence for maintenance of an acceptable quality of life, and the financial burden of PST was increasing.

His PCP contacted the VA Puget Sound **SU**pporting **P**rimary care **P**roviders in **O**pioid **R**isk reduction and **T**reatment (SUPPORT)—a National Center for Patient Safety-funded Patient Safety Center of Inquiry—to assist with evaluation for OUD. One week after the clinic visit, the SUPPORT center social worker assessed the patient and found that he met at least three Diagnostic and Statistical Manual of Mental Disorders, Fifth Edition, criteria for OUD: unsuccessful efforts to cut down use; a great deal of time spent in activities necessary to obtain and use opioids; and the withdrawal symptoms described above following cessation of use. After receiving detailed recommendations for initial dosing and titration from an experienced BUP prescriber through SUPPORT, the PCP prescribed buprenorphine-naloxone (BUP-NLX) for a home initiation 10 days after the initial visit. The patient discontinued all PST and oxycodone usage. Initial withdrawal symptoms started approximately 24 h later. The starting sublingual (SL) BUP-NLX dose was 2–0.5 mg with option of titration of up to 8–2 mg daily (mg/d) for withdrawal symptoms. However, the patient continued to experience withdrawal symptoms of sweating, rhinorrhea, restlessness and irritability, after 48 h. These symptoms were only partially relieved with an increase in BUP-NLX dosage to 24–6 mg/d 7 days after initiation. Repeat UDS were positive for BUP, and negative for other opioids.

10 days after initiation, he no longer experienced opioid cravings on the 24–6 mg/d dosage. By taking the prescription in doses of 8–2 mg TID, his pain was well controlled throughout the day. However, for the next month, he noticed flushing and anxiety for approximately 30 min after taking each dosage of BUP-NLX. Symptom support with clonidine for flushing and hydroxyzine for anxiety was not effective. Out of concern for intolerance to the BUP-NLX combination product, his PCP trialed BUP monotherapy at the same dose of BUP. Flushing after dosages abated following this medication switch. Because the patient’s initial goal was to use BUP to manage withdrawal and subsequently to discontinue all opioids, he requested to taper the dose of BUP to 2 mg TID. Trials of taking lower daily dosing than 6 mg led to increased pain and opioid cravings, so the 2 mg TID dosage of BUP was continued for the next 24 months. Voluntary UDS has only been positive for BUP. The patient felt liberated from the emotional, financial, and physical burden of PST dependence.

## Discussion

Our case highlights an atypical method of opioid use, drinking PST, leading to OUD. Recent studies suggest that PST use is increasing in the United States (U.S.). An analysis of cases of U.S. poppy consumption reported to the National Poison Data Systems and the Food and Drug Administration from 1968 to 2018 reported 64 cases of intentional PST consumption [6]. There was a trend towards increased intentional use of poppy seed products from 2000 to 2018. There are reports of PST use in other countries as well. Among patients surveyed in a New Zealand opioid addiction clinic in 2007, 46% of patients reported past consumption of PST [7], and among those surveyed in a United Kingdom addiction treatment center in 1990, 93% of patients reported consumption of PST [8].

The high concentration of morphine in PST leads to risk for overdose and OUD. PST use is directly associated with at least five U.S. fatalities in the twenty-first century [6], and there are six case reports of OUD developing from routine use of PST (Table 2). All cases of OUD were evaluated and treated in addiction treatment centers, and there are no published cases of home initiation of buprenorphine, nor of evaluation and treatment in primary care.

**Table 2:** Case reports of PST-related OUD

Patient	Age (years)	Gender	PST Consumption^a^	Initiation MOUD Dose	Maintenance MOUD Dose	Outcome
Case 1 [9]	37	M	14 pods^a^/day	Methadone 30 mg/d	Methadone 30 mg/d	Not reported
Case 2 [2]	26	M	4 kg seeds/day	Morphine 60 mg BID	Morphine 20 mg BID	Sustained abstinence
Case 3 [10]	43	F	5–6 kg seeds/week	BUP 4 mg/d	BUP 16 mg/d	Less than monthly use
Case 4 [10]	26	M	3 kg seeds/day	Methadone 30 mg/d	Methadone 110 mg/day	Sustained abstinence
Case 5 [11]	82	F	1–2 liters^a^/day	BUP 0.4 mg/d	BUP 0.8 mg/d	Sustained abstinence
Case 6 [12]	42	M	Unknown^a^	BUP-NLX 16–2 mg/d	BUP 18 mg/d	Sustained abstinence
Case 7^b^	47	M	2–4 cups/day	BUP-NLX 8–2 mg/d	BUP 6 mg/d	Sustained abstinence

Summary of existing case reports of PST-related OUD highlighting consumption, treatment with MOUD, and outcomes

F, female; M, male; BID, twice a day

^a^Did not quantify amount of poppy seeds used

^b^Case 7 is the patient described in this report

Our patient’s prolonged withdrawal symptoms suggest that PST withdrawal may be challenging to manage. Two other case reports of PST withdrawal observed in addiction treatment units described prolonged and severe symptoms [11, 12]. In addition to the known symptomatology of morphine and codeine withdrawal, discontinuing PST involves clearance of other opioid (thebaine) and non-opioid (papaverine, noscapine) substances whose physiologic effects are poorly understood [1, 3].

The patient’s improved tolerability to BUP monotherapy is similar to a case [12] in which a patient with PST-related OUD better tolerated BUP than the BUP-NLX combination. In that case, the patient’s urine was consistently positive for morphine after initiation of BUP-NLX, and subsequently negative while taking BUP, suggesting that precipitated withdrawal while on BUP-NLX may have led to the patient’s perception of improved tolerability to monotherapy. There is no evidence in pharmacokinetic studies that BUP-NLX has higher risk of precipitated withdrawal due to naloxone SL absorption compared to BUP [13, 14]. We had no suspicion of ongoing PST or opioid use while our patient took BUP-NLX given negative urine drug screening. It is possible that our patient had acutely worsened anxiety from withdrawal of either oxycodone or PST, and there was a temporal association with improvement of anxiety 6 weeks into treatment when the switch to BUP monotherapy occurred. In another case report [10], a patient had increased anxiety when he discontinued PST and started methadone. Providers prescribed venlafaxine prescription for control of symptoms. Monitoring for the effects of opioid abstinence on mental health, even while patients take MOUD, is important to help patients to engage in treatment.

The misinterpretation of the patient’s prior UDS results, specifically that a positive result for opiates was thought by clinicians to be due to the patient’s oxycodone use, was a missed opportunity to identify the patient’s PST use at an earlier point in his care. Prior research suggests that many providers who order UDS have limited understanding of opioid metabolism and the cross-reactivity of immunoassays, and misinterpretation of urine drug tests is common [15]. Expansion of UDS interpretation support programs [16] may help to improve provider interpretation of complicated UDS results for opioids.

There are many parallels between PST and kratom, an herbal supplement derived from tree leaves containing alkaloids that are opioid receptor agonists. Like PST, kratom is commercially available from online and in-store vendors, and is not regulated by the FDA. Consumption of kratom through brewed teas, powders, or pills produces analgesic effects, and there are increasing cases of OUD developing from routine use, as well as cases of individuals using kratom as treatment for OUD from other opioids. With an estimated 0.7% annual use in the U.S. adult population [17], kratom is likely more widely consumed than PST in the U.S., but treatment guidelines for OUD resulting from either substance do not exist. Due to uncertainties in the relative strength of opioids consumed from these substances, it may be difficult for the treating clinician to determine a sufficient dosage for initiation of BUP, which may explain the varied BUP initiation doses reported in Table 2. There may be uncertainty around maintenance doses as well, as prior case reports indicate varied BUP maintenance dosing. In a recent analysis of patients with OUD from kratom [18], those with higher self-reported kratom use at baseline tended to require a higher maintenance dose of BUP to manage OUD. Our patient initially needed a high dosage of BUP to manage withdrawal symptoms, but was later able to tolerate 6 mg daily during sustained remission. Symptom-directed titration of BUP, along with short interval follow-up, were the keys to successful dosing in our case. Given the variability in initiation and maintenance dosages, it is important that withdrawal symptoms are monitored closely with results used to inform adjustments to BUP dosing.

This case encapsulates the important progress being made for the treatment of patients with OUD in the primary care setting. Historically, patients with OUD could only access MOUD through federally regulated Opioid Treatment Programs. With the substantial increase in OUD largely a result of increased U.S. opioid prescribing in the past several decades [19], these facilities have been unable to keep pace with the high demand for MOUD [20]. Congress passed the Drug Addiction Treatment Act of 2000 (DATA 2000), which allowed qualified providers to prescribe BUP and naltrexone across various healthcare settings, including primary care [21]. These medications effectively treat OUD by reducing or eliminating illicit substance use and lowering risk of fatal and non-fatal overdose, and are safe medications [22] for prescribing by a general practitioner. A shift that further facilitated prescribing in primary care was the introduction of home initiation of BUP [23]. Therefore, a variety of care models now exist to encourage PCPs to diagnose and to treat OUD [24].

The SUPPORT center at the VA Puget Sound Medical Center, funded by the Veterans Health Administration’s National Center for Patient Safety [25], is a unique model of care which offers PCPs a range of options for prescribing MOUD: (1) open-access consultation with a social worker specializing in OUD and an experienced BUP provider, who give recommendations to help the PCP to diagnose OUD and to initiate BUP independently; (2) initial diagnosis and treatment with BUP by the SUPPORT team, with transition of prescription to the PCP when the BUP dosage has stabilized; and (3) initiation and indefinite prescribing of BUP by the SUPPORT team if the PCP feels uncomfortable with managing BUP.

Although recently waivered by the Drug Enforcement Agency to prescribe BUP, the PCP in this case had never prescribed MOUD, and yet felt empowered to initiate BUP-NLX using the consultation model described above. The long-term relationship that existed between the PCP and the patient, the availability of help from the SUPPORT team, and the option to take BUP through PCC without the burden and stigma of an addiction treatment program were important components to success. Our model of care allows PCPs with limited experience with MOUD to manage OUD in primary care.

## Conclusion

PST, which can be made through purchase of readily available poppy pods, carries risk for development of OUD and overdose. Policymakers should consider interventions to regulate the sale of these products. Primary care clinicians, with the help of office-based opioid therapy support teams, can be empowered to identify and treat OUD with BUP in primary care.

## Data Availability

No data was collected for the purposes of this study. The information used for this case report comes from the patient’s record.

## Abbreviations

PST: Poppy seed tea
OUD: Opioid use disorder
MOUD: Medications for opioid use disorder
SUPPORT: SUpporting Primary care Providers in Opioid Risk reduction and Treatment
MME: Morphine milligram equivalents
PCC: Primary care clinic
PTSD: Post-traumatic stress disorder
TID: three times a day
mg/d: Milligrams per day
h: Hours
UDS: urine drug screen
PCP: Primary care provider
BUP-NLX: Buprenorphine-naloxone
BUP: Buprenorphine
SL: Sublingual
BID: twice a day
